# Wearable Electrocardiogram Technologies for the Early Detection of Acute Coronary Syndromes

**DOI:** 10.1016/j.jacasi.2026.05.015

**Published:** 2026-07-01

**Authors:** Yue Wang, Bing-Yan Yu, Wai Chak Wong, Chor-Cheung Tam, Chun-Ka Wong, Hung-Fat Tse

**Affiliations:** a Cardiology Division, Department of Medicine, Li Ka Shing Faculty of Medicine, The University of Hong Kong, Hong Kong, China; b Heart and Vascular Center, The University of Hong Kong-Shenzhen Hospital, Shenzhen, China; c Hong Kong-Guangdong Stem Cell and Regenerative Medicine Research Centre, The University of Hong Kong and Guangzhou Institutes of Biomedicine and Health, Hong Kong, China; d Center for Translational Stem Cell Biology, Hong Kong Science Park, Shatin, New Territories, Hong Kong, China; e HKUMed Laboratory of Cellular Therapeutics, University of Hong Kong, Hong Kong, China; f State Key Laboratory of Pharmaceutical Biotechnology, The University of Hong Kong, Hong Kong, China; g Advanced Biomedical Instrumentation Centre, Hong Kong Science Park, Shatin, New Territories, Hong Kong, China; h Centre for Regenerative Medicine and Health, Hong Kong Institute of Science & Innovation, Chinese Academy of Sciences, Hong Kong Science Park, Shatin, New Territories, Hong Kong, China

**Keywords:** acute coronary syndrome, artificial intelligence, digital health, electrocardiography, wearable devices

## Abstract

Ischemic heart disease remains the leading cause of cardiovascular mortality globally, with acute coronary syndrome (ACS) representing its most critical clinical manifestation. Although the standard 12-lead electrocardiogram is the diagnostic cornerstone for ACS, its utility is limited by the intermittent nature of recording and the requirement for clinical infrastructure, potentially leading to delayed diagnosis or missed atypical acute ischemic events. Advances in sensor technology have enabled the development of wearable electrocardiogram devices capable of continuous, ambulatory monitoring. This review examines the current landscape of wearable technologies for ACS detection, categorizing them into smartwatches, handheld monitors, patch-based systems, and textile-based garments. We evaluate the diagnostic performance of these modalities, highlighting that single-lead devices offer convenience, whereas multilead configurations are essential for the accurate localization of acute ischemia and the reduction of false negatives. Furthermore, we address critical challenges impeding clinical adoption, including signal fidelity amidst motion artifacts, the lack of large-scale validation studies, and challenges in integrating data into clinical workflows. Finally, we discuss future directions, emphasizing the role of multimodal sensing—combining electrophysiology with biochemical or mechanical sensors—and artificial intelligence in ushering in a new era of personalized monitoring for ACS.

## Highlights

• Wearable ECG devices enable early warning of acute ischemia.
• Multilead ECG improves diagnostic sensitivity over single-lead devices.
• AI and multimodal sensing represent the future of precision monitoring.
• Large-scale validation is essential for wearables in clinical practice.

Myocardial ischemia represents the pathophysiological cornerstone of ischemic heart disease, a condition characterized by a critical imbalance between myocardial oxygen supply and metabolic demand. Although primarily driven by obstructive coronary artery disease and the subsequent limitation of coronary blood flow by atherosclerotic plaque, ischemia can also result from microvascular dysfunction or vasospasm. The spectrum of this imbalance is broad, encompassing both subclinical presentations—such as silent ischemia, which is optimally detected through functional stress imaging (eg, single-photon emission computed tomography, positron emission tomography, cardiac magnetic resonance imaging, or stress echocardiography)—and overt clinical manifestations, including stable angina and acute coronary syndrome (ACS). Although functional imaging remains the gold standard for identifying subclinical ischemia, the scope of this review focuses explicitly on the clinical presentations of ACS: unstable angina, non–ST-segment elevation myocardial infarction, and acute coronary occlusion leading to ST-segment elevation myocardial infarction (STEMI). Among these, STEMI constitutes the most severe manifestation, associated with transmural cardiomyocyte necrosis, precipitous electrical instability, and a substantial risk of sudden cardiac death.^1^^,^^2^ The global burden of ischemic heart disease is staggering, remaining the leading cause of mortality worldwide^3^ and accounting for over 4 million deaths annually in Europe and Northern Asia alone.^4^ Consequently, the imperative for early recognition and timely reperfusion in ACS—adhering to the “time is muscle” paradigm—is critical to limiting infarct size, preserving left ventricular function, and improving survival rates.^5^

The diagnostic cornerstone for ACS remains the standard 12-lead electrocardiogram (ECG). By providing a comprehensive spatial assessment of cardiac electrical activity,^6^^,^^7^ the 12-lead ECG allows clinicians to detect repolarization abnormalities—such as ST-segment deviation, T-wave inversion, and pathological Q waves—that are pathognomonic of acute ischemia.^8^ When integrated with clinical presentation, cardiac biomarkers (specifically, high-sensitivity troponins), and imaging modalities, the ECG guides the decision pathway for urgent revascularization. However, despite its established clinical value, the standard 12-lead ECG is bound by significant operational and temporal constraints.^9^ It is inherently a static diagnostic tool, capturing only a brief, 10-second snapshot of cardiac activity. This intermittent nature renders it ill-suited for detecting transient acute ischemic episodes or dynamic ST-segment changes that occur prior to hospital arrival. Furthermore, the requirement for specialized equipment and trained personnel restricts its availability primarily to clinical settings, creating a “diagnostic gap” between symptom onset and medical contact.

This gap is exacerbated by barriers to accessibility and patient behavior. Prehospital delays remain a major determinant of outcomes, with studies indicating that more than 16% of male and 30% of female patients with myocardial infarction (MI) do not seek timely emergency medical attention, often because of symptom ambiguity or lack of access to immediate diagnostics.^10^ In resource-limited settings, this delay is further compounded by the scarcity of health care infrastructure. These limitations underscore a critical unmet need for diagnostic solutions that are accessible, continuous, and capable of real-time monitoring outside the hospital environment. In response to this need, the convergence of advances in sensor technology, miniaturization, and wireless communication has catalyzed the development of wearable ECG devices. By transitioning cardiac surveillance from the clinic to the community, these devices aim to overcome traditional diagnostic barriers, offering the potential for long-term ambulatory monitoring and the earlier detection of acute coronary events.

## Landscape and Clinical Validation of Wearable ECG Modalities

The landscape of ambulatory cardiac monitoring has undergone a radical transformation, moving beyond the cumbersome, wired architecture of traditional Holter monitors toward miniaturized, wireless, and user-friendly form factors.^11^ These devices can be categorized into 4 distinct phenotypes based on their design, electrode configuration, and intended clinical utility: smartwatches, handheld ECG monitors, patch-based systems, and textile-based garments. Each category presents a unique compromise between ease of use, duration of monitoring, and the density of electrophysiological data available for the detection of ACS. Evaluating their utility requires examining both their underlying technical capabilities and their clinical validation simultaneously.

### Smartwatches

Smartwatches represent the most ubiquitous form of wearable technology, having evolved from consumer fitness trackers into sophisticated diagnostic platforms. The fundamental innovation in modern smartwatches is the integration of dry-electrode ECG sensors, which complement photoplethysmography-based rhythm monitoring.^12^^,^^13^ The standard operation uses a single-lead configuration: One electrode is situated on the dorsal aspect of the watch case (in contact with the wrist), whereas a second electrode is integrated into the crown or bezel.^14^ To acquire an ECG, the user creates a closed electrical circuit across the thorax by touching the crown with a finger from the contralateral hand, generating a bipolar lead I equivalent.

Although originally designed and highly validated for arrhythmia detection, these devices are increasingly positioned as tools for the early detection of ACS. Illustrative case reports have demonstrated that a single-lead smartwatch ECG can occasionally identify severe ischemic events, such as left anterior descending artery stenosis in a patient with stable angina.^15^ However, clinical validation studies reveal severe limitations for broad ACS screening. Because lead I captures a limited spatial view of the heart's electrical activity, studies report sensitivities as low as 34% for detecting acute ST-segment deviations, although specificity approaches 100%.^16^ This discrepancy indicates that although a positive ST-elevation result is reliable, a significant proportion of acute ischemic events—particularly those involving the inferior or posterior walls—will be missed if relying solely on standard single-lead recordings.

To overcome this spatial limitation, advanced user protocols have been validated wherein the watch is sequentially placed on specific abdominal and precordial locations (positions V_1_-V_6_) while maintaining ground contact.^17^^,^^18^ This “phantom lead” approach allows for the reconstruction of Einthoven and Wilson-like leads.^19^^,^^20^ Clinical validation studies involving patients with STEMI and non–ST-segment elevation myocardial infarction demonstrate strong agreement between these reconstructed multilead smartwatch tracings and standard 12-lead ECGs for key diagnostic features, significantly improving the detection of abnormal ST-T changes.^21^^,^^22^ In cases of acute anterior MI, this method accurately identified ST-segment elevation in the Wilson-like leads and correctly inferred coronary occlusion.^23^ Moreover, patient-operated precordial lead recording has shown high feasibility, with 98% of users able to complete the procedure successfully.^18^ Nevertheless, practical limitations persist; although multilead configurations improve diagnostic accuracy, they necessitate complex, active user participation that may be unfeasible for individuals experiencing ongoing, severe chest pain.

### Handheld ECG devices

Handheld ECG monitors occupy a distinct niche as “on-demand” diagnostic tools designed for rapid assessment in outpatient or home settings. Unlike passive wearables, these devices require active patient engagement but offer superior baseline lead configurations.^24^ A representative example, the KardiaMobile 6L (AliveCor), uses a triangular electrode arrangement: 2 stainless steel electrodes on the superior surface for contact with the thumb and a third electrode on the inferior surface for contact with the left knee or ankle.^25^ By establishing 3 points of contact, the device replicates Einthoven's triangle to record the 6 standard limb leads simultaneously, and it can approximate precordial leads via off-label placement on the chest wall.^26^^,^^27^

Clinical validation of these handheld devices demonstrates high diagnostic accuracy for detecting acute ischemic changes, making them highly practical for rapid triage. A study evaluating a handheld device's integrated multilead system reported approximately 87% sensitivity and 96% specificity for acute coronary occlusion.^28^ Further clinical validation confirmed an overall diagnostic accuracy of 77.4% for myocardial supply ischemia, which was comparable to the 72.8% accuracy observed with traditional 12-lead ECGs in the same cohort. The ability to reconstruct a 13-lead ECG that combines standard leads with a dynamic ST vector further enhances the localization of acute ischemic regions.^29^ While highly suitable for emergency assessments and automated outpatient follow-up, significant limitations exist. The diagnostic effectiveness of handheld monitors is constrained during transient, asymptomatic ischemic episodes given their intermittent recording nature, and their accuracy relies heavily on correct anatomical lead placement by the user.

### Patch-based ECG devices

Patch-based systems have emerged as the standard of care for extended ambulatory monitoring, effectively replacing traditional Holter monitors in many clinical workflows. These devices integrate flexible electronics, distinct leadless designs, and adhesive hydrogels into a compact, water-resistant unit capable of continuous recording for up to 14 days with minimal user intervention.^30^^,^^31^ The primary advantage of patch-based systems is their ability to acquire continuous data, facilitating the detection of transient or silent ischemic events that intermittent recordings (like smartwatches or handhelds) often miss. However, most commercially available patch devices are currently limited to single-vector recording,^32^^,^^33^ which restricts the precise anatomical localization of cardiac abnormalities.

To address this, the research frontier is rapidly advancing toward dual-modal patches that integrate electrophysiological sensing with biochemical detection. A recently reported wearable patch incorporates surface-enhanced Raman scattering microneedles, enabling simultaneous monitoring of ECG signals and interstitial fluid biomarkers (eg, troponin, myoglobin).^34^ Preclinical validation of this dual-modal system in a rat model of acute MI demonstrated that the patch could detect ST-segment elevation within 20 minutes of coronary occlusion and elevated myocardial injury biomarkers within 50 minutes.^34^ Although these innovations confirm the capacity of the patch for early, multimodal detection, clinical validation in human patients for ACS (rather than arrhythmia) remains insufficient, and challenges regarding skin irritation during prolonged wear persist.

### Textile-based ECG garments

Textile-based ECG systems, or “smart garments,” use conductive yarns (eg, silver-coated nylon or stainless steel threads) woven directly into the fabric structure to function as dry electrodes. These systems integrate compact processing modules for signal amplification and wireless transmission, aiming to combine the comprehensive spatial coverage of a 12-lead ECG with the comfort of daily clothing.^35^ Unlike adhesive patches or watches, textile systems have the unique potential to provide continuous, full 12-lead monitoring without skin irritation.

Currently, textile-based garments remain largely in the stage of technical validation. Research demonstrates their ability to acquire clinically acceptable signal quality over extended periods,^36^^,^^37^ and their extended wearability has been shown to improve the detection rates of intermittent conditions like atrial fibrillation (AF).^38^ Theoretically, this capacity for prolonged, comprehensive monitoring is ideal for identifying acute transient ischemic episodes. However, significant technical and clinical barriers remain. The signal quality of textile electrodes is highly sensitive to the stability of the electrode-skin interface; motion artifacts, changes in pressure, and variations in skin moisture (sweat)^38^ can significantly alter impedance and lead to baseline wander. Furthermore, ensuring the durability and washfastness of the conductive components remains a material science challenge. To date, these systems lack robust peer-reviewed evidence validating their diagnostic accuracy, specifically for ACS.

The diagnostic performance and key characteristics of the primary wearable ECG device categories discussed are systematically summarized in Tables 1 and 2.

**Table 1:** Diagnostic Performance of Wearable ECG Devices in Detecting Acute Coronary Syndrome

Device Type First Author	Patients	Leads Recorded	Outcome	Testing Efficacy
Smartwatch				
Caillol et al^16^	256	Single lead	ACS with ST-segment abnormalities	Sensitivity: 0.34; specificity: 1.00
Ploux et al^21^	260	4 leads	Ischemia with abnormal ST-T	Sensitivity: 0.77; specificity: 0.92
Samol et al^19^	50	6 leads	Acute anterior MI	Sensitivity: 1.00; specificity: 1.00
Spaccarotella et al^22^	100	9 leads	MI (STEMI and NSTEMI)	STEMI—sensitivity: 0.93; specificity: 0.95 NSTEMI—sensitivity: 0.94; specificity: 0.92
Handheld ECG				
Van Heuverswyn et al^28^	64 patients; 5,011 self-recordings	3 leads	Acute coronary occlusion	Sensitivity: 0.87; specificity: 0.96
Van Heuverswyn et al^29^	110	13 leads	Acute myocardial ischemia	Accuracy: 0.77; sensitivity: 0.82
Patch-based ECG				
Yang et al^34^	Not available	Single lead with myocardial injury biomarkers	Acute MI	Data not available

The wide range of sensitivities and specificities summarized in this table underscores a central challenge in the field. The primary determinant of diagnostic sensitivity is the number of ECG leads acquired. Single-lead devices, although highly convenient and portable, generally offer insufficient diagnostic yield for acute ischemia and are unsuitable for rule-out purposes. Conversely, multilead devices demonstrate improved performance, particularly in detecting overt STEMI. However, their sensitivity for NSTEMI or unstable angina remains limited, influenced by factors such as device design, clinical context, and patient groups. Variability across studies is also affected by differences in reference standards, study methodologies, and patient selection. These findings highlight the urgent need for large-scale, standardized validation studies focusing on diverse patient groups and uniform endpoint definitions to better establish the clinical utility of wearable ECG technologies for ischemia detection.

ACS = acute coronary syndrome; ECG = electrocardiogram; MI = myocardial infarction; NSTEMI = non–ST-segment elevation myocardial infarction; STEMI = ST-segment elevation myocardial infarction.

**Table 2:** Summary Table of Device Types

Device Type	Key Features	Advantages	Limitations and Challenges	FDA Clearance
Smartwatch	1. Typically starts with single-lead (lead I)	1. High portability and accessibility	1. Low sensitivity (∼34%) with single-lead for ischemia	Cleared for ECG (AF detection, rhythm classification)—eg, Apple Watch (DEN180044); ischemia/ACS detection not indicated
	2. Multilead capable (eg, I, II, III, V_1_-V_6_) via user maneuver	2. Real-time monitoring and alerts	2. Multilead operation is cumbersome and time -consuming	
	3. User-initiated recording	3. High user acceptance and engagement	3. Not suitable for ruling out acute ischemia	
Handheld ECG	1. Simulated chest or dual-hand leads	1. Simple operation and rapid assessment	1. Only suitable for short-term, intermittent monitoring	Cleared for multilead recording and rhythm analysis—eg, KardiaMobile 6L (K211900); ischemia/ACS detection not indicated
	2. Some devices support 3+ leads (eg, KardiaMobile 6L)	2. Suitable for home use and patient self-monitoring	2. Likely to miss asymptomatic ischemia	
	3. Designed for quick, on-demand spot checks			
Patch-based ECG	1. Adhesive patch with embedded electrodes	1. Enables long-term, continuous data capture 2. Discreet and minimal hindrance to daily life 3. Can detect transient ischemic events 4. High potential for multimodal assessment (electrophysiological + biochemical)	1. Limited lead coverage (often single lead)	Cleared for continuous ambulatory monitoring (arrhythmia detection)—eg, Zio Patch (K121319); ischemia/ACS detection not indicated
	2. Ambulatory, continuous monitoring		2. Signal quality susceptible to motion artifacts during activity	
	3. Emerging integration with biomarkers		3. Lack of robust clinical validation specifically for acute ischemia detection	
Textile-based ECG garment	1. Conductive fabrics woven/embroidered into garments	1. Superior comfort for long-term use	1. Limited wash durability of conductive materials	No FDA clearance; investigational use only
	2. Capable of full 12-lead ECG monitoring	2. Comprehensive lead coverage akin to standard ECG	2. Highly susceptible to motion artifacts and influenced by body habitus and movement	
	3. Designed for comfort and prolonged wear	3. Ideal for continuous ambulatory monitoring	3. Currently lacks peer-reviewed validation for diagnosing acute ischemia	

ACS = acute coronary syndrome; AF = atrial fibrillation; ECG = electrocardiogram; FDA = Food and Drug Administration.

## Clinical Use Case Framework: Screening vs Diagnostic Confirmation

As wearable ECG technologies mature, their integration into clinical practice requires a clear framework that distinguishes between different intended use cases. The performance metrics of importance—and the clinical consequences of device failure—differ fundamentally depending on whether the technology is deployed for primary screening or diagnostic confirmation. Evaluating device performance without this clinical context limits the ability of health care systems to safely implement these tools. Table 3 maps the current wearable modalities against these 2 distinct clinical paradigms.

**Table 3:** Clinical Use Case Framework for Wearable ACS Detection

Clinical Parameter	Primary Screening/Early Warning	Diagnostic Confirmation (Symptom-Driven)
Target patient group	Asymptomatic, high-risk individuals (eg, prior MI, severe CAD).	Symptomatic individuals (eg, experiencing acute chest pain or angina equivalents).
Intended goal	Detect silent or early onset acute ischemic changes before symptom recognition.	Triage acute symptoms to confirm or rule out an evolving ACS event.
Priority metrics	Sensitivity and negative predictive value	Specificity and positive predictive value
Clinical risk of failure	False negatives: Missed ACS, delayed revascularization, false sense of security, increased mortality.	False positives: Unnecessary emergency medical services visits, alarm fatigue, catheterization laboratory misactivations, patient anxiety.
Single-lead devices (eg, standard smartwatch)	Inadequate: High false negative rate (∼34% sensitivity) misses anatomically silent regions (eg, inferior/posterior walls).	Partial utility: Can “rule in” an event if ST-segment elevation is captured (high specificity) but cannot safely “rule out” ACS.
Multilead devices (eg, patches, smart textiles)	Required: Multivector spatial resolution is necessary to detect focal acute ischemic changes and minimize false negatives.	Required for “rule out”: Comprehensive spatial views are needed to confidently reassure patients and prevent unnecessary ED visits.

ACS = acute coronary syndrome; CAD = coronary artery disease; ED = emergency department; MI = myocardial infarction.

### Primary screening and early warning

In a primary screening or early warning context, wearable devices are typically deployed continuously in high-risk, asymptomatic individuals to detect the onset of ACS before severe symptoms prompt medical attention. In this paradigm, high sensitivity and high negative predictive value are paramount. The primary clinical risk is a false negative: missing an evolving ACS event, which leads to delayed revascularization, irreversible myocardial necrosis, and increased mortality.

Evaluated against this standard, single-lead smartwatches are currently inadequate for screening. As noted previously,^16^ the ∼34% sensitivity for ST-segment deviation inherent to a single-lead view means that roughly two-thirds of acute ischemic events would be missed, providing a false sense of security to vulnerable patients. Therefore, for early warning applications, continuous multilead systems—such as advanced adhesive patches or smart textiles with synthesized 12-lead capabilities—are strictly required to achieve the necessary spatial coverage and sensitivity thresholds.

### Diagnostic confirmation (symptom-driven)

Conversely, in a diagnostic confirmation context, wearables are used by patients to record an ECG during an acute symptomatic episode (eg, chest pain or angina equivalents) to help triage the need for emergency care. In this scenario, high specificity and high positive predictive value become the priority metrics. Because the pretest probability of ACS is already elevated by the presence of symptoms, the primary clinical risk shifts to false positives. Widespread deployment of a device with low specificity in this context would result in severe “alarm fatigue,” overwhelming emergency departments with unnecessary visits, triggering inappropriate invasive testing, and generating significant patient anxiety.

In this symptom-driven paradigm, single-lead smartwatches hold more clinical utility. Because their specificity for detecting gross ST-segment elevation often approaches 100%, a positive finding during a chest pain episode can rapidly “rule in” an event and accelerate emergency medical services dispatch. However, given their limited sensitivity, single-lead devices cannot safely “rule out” ACS if the tracing is normal. To safely rule out acute ischemic changes and prevent unnecessary hospital visits, multilead configurations (such as user-directed multilead smartwatch maneuvers or multielectrode handheld devices) are required to provide comprehensive diagnostic reassurance.

## Challenges and Future Directions

Despite the rapid evolution of sensor technology, the transition of wearable ECG devices from consumer wellness trackers to clinically validated instruments for ACS detection faces significant hurdles. These challenges—such as signal fidelity, anatomical coverage, and clinical workflow integration—must be addressed to realize the full potential of ambulatory monitoring for acute ischemic events.

### Signal fidelity and the electrode-skin interface

The most immediate barrier to ambulatory monitoring is ensuring signal fidelity amidst the dynamic noise floor of daily life. Unlike standard resting ECGs, wearable devices must contend with significant motion artifacts, electromyographic noise, and baseline wander that can obscure subtle repolarization abnormalities. Traditional wet Ag/AgCl electrodes, although being the clinical gold standard, are prone to desiccation over time, leading to impedance mismatches that mimic ST-segment shifts.^39^ To overcome this, future device development must focus on dry electrode technologies using advanced nanomaterials—such as graphene-coated textiles, silver nanowire networks, or carbon nanotube composites. These materials offer superior conformability to skin microtopography, maintaining stable contact without the need for conductive gels. Critically, distinguishing motion-induced artifacts from true acute ischemic changes is an algorithmic challenge. Standard linear filtering can inadvertently distort the low-frequency components of the ST-segment, rendering it unsuitable for this task. This has spurred the development of more sophisticated techniques. Early approaches, such as empirical mode decomposition and wavelet transforms, offered significant improvements by isolating artifacts in the time-frequency domain without compromising waveform morphology.^40^ More recently, deep learning–based denoising approaches, such as convolutional neural networks (CNNs), autoencoders,^41^^,^^42^ redundant convolutional encoder-decoder networks,^43^ and generative adversarial networks (GANs),^44^ can learn to separate true cardiac signals from noise in the time-frequency domain. Studies have demonstrated that these methods can effectively suppress motion artifacts, electromyographic noise, and baseline wander in ambulatory ECG recordings, thereby improving signal-to-noise ratio and preserving diagnostic waveform morphology. The integration of such artificial intelligence (AI)–powered denoising algorithms directly into wearable devices or cloud-based platforms holds the potential to transform raw, artifact-contaminated data into clinically interpretable signals, a critical step toward reliable ACS detection in real-world settings.

### Lead coverage and spatial resolution

Diagnostic accuracy for localizing acute ischemic territories is intrinsically limited by the spatial resolution of current wearables. Most commercial devices rely on a single bipolar lead (lead I), which is orthogonal to the electrical vector of the inferior wall. Consequently, these devices are effectively “blind” to acute ischemia in the right coronary artery and left circumflex territories, risking the omission of regional ischemia.^45^ Addressing this limitation requires reconciling the inherent trade-off between wearability and diagnostic completeness. One promising approach is derived electrocardiography and lead synthesis. By using reduced-lead sets such as the EASI system or Frank orthogonal leads, computational models can mathematically reconstruct a 12-lead ECG.^46^^,^^47^ More recently, the same reduced-lead configurations have been combined with data-driven deep learning architectures, particularly GANs and CNNs. Grande-Fidalgo et al^48^ reported root mean square (RMS) errors below 13 μV using artificial neural networks for lead reconstruction, underscoring the technical maturity of this approach. Zhan et al^49^ demonstrated that GAN-based reconstruction could operate at the level of single heartbeats, generating complete 12-lead signals from a single-beat input captured at lead I with correlation coefficients exceeding 0.74 at the beat level. Yoon and Joo^50^ demonstrated that GAN-generated 12-lead ECGs could achieve diagnostic classification performance surpassing that of the original recordings. Similarly, Safdar et al^51^ developed translational algorithms that attained 82% accuracy for single-lead analysis by leveraging 12-lead training data. These innovations offer a potential pathway to reconcile the tension between reduced even single-lead wearability and the diagnostic completeness of the standard 12-lead configuration.

Despite these technical advances, the clinical utility of lead synthesis for comprehensive cardiac assessment remains uncertain. Using lead I alone or combined leads I and II as input, Presacan et al^52^ demonstrated that GAN-based reconstruction exhibits a regression-to-the-mean effect, biasing amplitudes toward patient group averages rather than preserving patient-specific variability. This finding raises concerns about whether current methods can retain the individualized spatial information essential for nuanced clinical interpretation, particularly for ACS, a condition heavily dependent on detailed morphologic patterns across multiple leads. Furthermore, it is imperative to acknowledge a critical validation gap in this literature. Although these reconstruction algorithms are highly validated for signal fidelity and arrhythmia classification, they have not yet been rigorously validated for the detection of precise, dynamic ST-segment deviations specifically required for ischemia diagnosis. Because ACSs often present with highly localized, subtle spatial changes, proving that generative models can accurately infer ischemic morphology—rather than just normal electrophysiology—remains a major frontier for future research before these algorithms can be safely implemented for ACS triage.

### The regulatory gap and clinical validation

A distinct regulatory divergence exists between arrhythmia and ACS detection. Although numerous devices are Food and Drug Administration–cleared for AF, automated acute ischemia detection remains uncleared because of the high risks associated with false positives and negatives.^53^ This risk stems from the fundamental nonspecificity of ambulatory ST-segment changes, which may result from tachycardia, electrolyte imbalances, or early repolarization, rather than acute ischemia. To achieve clinical utility, validation studies must demonstrate a device's ability to discriminate ischemic ST-segment depression from these benign mimics with high specificity. However, traditional fixed ST-segment thresholds may fail to account for significant interindividual variability, leading to inaccuracies. This inherent limitation underscores the need for patient-specific approaches that can adapt to an individual's unique electrophysiological patterns. Personalized baselines offer a potential solution by establishing individual-specific reference thresholds. Integrating personalized baselines with continuous ST-segment monitoring may help distinguish pathological acute ischemia from benign physiological variations and reduce false alarms. Ultimately, rigorous, prospective, multicenter trials are urgently needed to evaluate these devices against standardized clinical endpoints, such as angiographic stenosis or high-sensitivity troponin levels. Such studies are prerequisites for establishing definitive sensitivity, specificity, and ambulatory acute ischemic thresholds, which likely differ significantly from standard resting ECG criteria.

### AI in signal processing, lead reconstruction, and multimodal integration

As discussed in previous sections, AI has demonstrated 2 core capabilities in wearable ECG analysis. First, deep learning algorithms—including CNNs, autoencoders, and GANs—effectively remove motion artifacts and baseline wander, thereby enhancing signal fidelity.^40^, ^41^, ^42^, ^43^, ^44^ By effectively mitigating these artifacts, AI-driven noise reduction directly addresses one of the primary physical limitations of textile and patch-based wearables identified earlier in this review.

Beyond signal clarification, AI offers a transformative algorithmic solution to the fundamental trade-off between patient wearability (which favors fewer leads) and diagnostic completeness (which requires multiple leads). Instead of requiring the user to physically move a single-lead device sequentially across different body sites to capture spatial data, several recent studies have demonstrated the feasibility of reconstructing multiple standard leads (eg, II, III, aVF, V_1_-V_6_) from a single-lead input—such as one captured at the wrist—using GANs and CNNs.^48^^,^^50^^,^^51^

Even with pristine signal processing and lead reconstruction, single-modality ECG monitoring has inherent limitations. The ischemic cascade begins with metabolic disturbances and mechanical dysfunction before electrical changes manifest, meaning ECG alone may miss early events. Multimodal sensing addresses this gap by integrating complementary signals. Mechanical sensing via seismocardiography (SCG) can detect wall motion abnormalities that precede ST-segment changes. Biochemical sensing using microneedle arrays enables rapid detection of troponin or myoglobin in interstitial fluid. Recent advances in wearable technology have demonstrated robust hardware platforms capable of integrating SCG with ECG for high-fidelity signal acquisition during motion, enabling real-time diagnosis of MI and other cardiac conditions.^54^ By capturing the ischemic cascade—from early SCG-detected mechanical dysfunction to ECG repolarization abnormalities and then troponin release—such a multimodal system enables complementary assessment of acute ischemia via electrophysiological, mechanical, and biochemical pathways.

However, integrating diverse data streams creates analytical complexity beyond human capacity. AI, particularly deep learning models, offers a promising approach to fusing these inputs, with the potential to identify acute ischemic signatures that might not be apparent from any single modality alone. Beyond traditional classification, AI offers a pathway toward personalized monitoring through the emerging concept of a “digital twin.” A digital twin is a virtual representation of an individual's cardiovascular physiology, built from their unique, longitudinal multimodal data such as ECG, photoplethysmography, heart rate variability, sleep patterns, physical activity, and clinical information.^55^ These models are continuously updated over time through AI algorithms. Recent methodological work has demonstrated the feasibility of constructing accurate and efficient cardiac digital twins directly from surface ECGs.^56^ Building on the concept of personalized baselines introduced in previous sections, advances in generative AI–enabled digital twins have emphasized the construction of dynamic, individualized physiological baselines through the integration of multimodal data.^56^, ^57^, ^58^ By establishing a personalized baseline of cardiac electrical activity, the model can identify subtle, patient-specific alterations indicative of acute coronary events that might otherwise be missed by patient group–level criteria. Furthermore, continuous integration of multimodal data enables the construction of a dynamic patient profile capable of predicting impending clinical events and patient trajectories before they become apparent.^59^ Although the scalability and clinical impact of such solutions remain to be fully demonstrated, this approach has the potential to transform monitoring, treatment, and personalization of care in cardiology.

Although much of the challenge in wearable cardiology revolves around capturing these complex morphological changes such as ST-segment deviations, a major and rapidly expanding frontier lies in the screening and longitudinal management of AF. Wearables have already demonstrated significant clinical utility in large-scale, patient group–level AF screening, enabling the early detection of subclinical or paroxysmal arrhythmias that might otherwise remain undetected until a cryptogenic stroke occurs.^60^^,^^61^ Furthermore, continuous ambulatory monitoring via wearables is increasingly being integrated into postprocedural care, particularly for the detection of AF recurrence after catheter ablation. The ability to passively monitor a patient's rhythm burden over extended periods, without the strict need for surgically implantable loop recorders, offers a noninvasive, highly accessible paradigm for postablation surveillance. This continuous data stream is vital for assessing procedural success and guiding subsequent decisions regarding anticoagulation and rhythm control therapies.^62^

Finally, although the diagnostic performance data presented in this review primarily focus on ACS, the convergence of multimodality and AI also holds theoretical potential for detecting subclinical myocardial ischemia—transient, asymptomatic episodes that are currently diagnosed using functional stress imaging modalities such as single-photon emission computed tomography, positron emission tomography, cardiac magnetic resonance imaging, or stress echocardiography.^63^ This represents a critical logical extension for wearable technology. However, translating this potential into clinical practice will require rigorous validation studies that directly compare wearable-derived signals against these established reference standards to establish definitive diagnostic accuracy in asymptomatic patients.

## Beyond Technology: Implementation and Equity Considerations

The preceding sections have detailed the technical and regulatory challenges that must be overcome to realize wearable detection of ACS. Yet, even if these hurdles are surmounted, a more fundamental question remains: For whom will this technology work? The transition from clinic-based to community-based monitoring, although promising, will only improve patient health if devices are actually used by patients, seamlessly integrated into health care systems, and accessible to all segments of society.^57^ This section examines these interconnected challenges across 3 levels: the individual user, the health care system, and the broader social context.

At the individual level, technological capability is futile without patient adherence. Complex device setups lead to poor compliance, particularly among the elderly persons most at risk for coronary artery disease.^33^ User-centric design must therefore prioritize “fit-and-forget” form factors with intuitive interfaces and minimal maintenance, ensuring that devices integrate effortlessly into daily life rather than becoming an additional burden. At the health care system level, seamless integration is essential to prevent “alarm fatigue.” Middleware platforms are required to filter raw data and transmit only actionable alerts to the electronic health record. Furthermore, adherence to interoperability standards, such as HL7 FHIR, is critical to ensuring that a smartwatch ECG recorded during a prehospital acute chest pain event is immediately viewable by the emergency physician upon the patient's arrival, facilitating rapid triage.^64^

At the societal level, the promise of wearable technology risks exacerbating existing health disparities if not deliberately designed for equity from the outset. These concerns extend beyond cost barriers and warrant dedicated consideration across multiple dimensions.

### The digital divide and access barriers

Wearable ECG devices are not standalone technologies; they require complementary infrastructure that may be unevenly distributed. Smartphone ownership, reliable internet connectivity, and digital literacy—prerequisites for most wearable devices—remain lower among older adults, lower socioeconomic groups, and certain racial and ethnic minorities.^65^ These patient groups paradoxically bear a disproportionately higher burden of cardiovascular mortality and often experience delayed presentation with ACS.^66^ Furthermore, the economic model of many wearables involves recurring costs—device subscription fees, cloud storage, or premium analytics services—that create financial barriers to sustained use. Without deliberate mitigation strategies, those who are at highest risk may be systematically excluded from the benefits of continuous monitoring for acute coronary events.

### Algorithmic fairness in AI-enabled ECG interpretation

As AI becomes increasingly integrated into wearable ECG analysis, the question of algorithmic fairness emerges as a critical equity concern. Deep learning models for ECG interpretation are typically trained on data sets that may not adequately represent racial, ethnic, or demographic diversity. Emerging evidence suggests that AI-ECG algorithms can exhibit differential performance across subgroups.^67^ If such algorithms are deployed in wearable devices without rigorous validation across diverse patient groups, they risk misclassifying acute ischemic changes—or missing them entirely—in under-represented groups. This could inadvertently widen, rather than narrow, disparities in ACS detection and outcomes. To date, few of the devices and algorithms reviewed in this article have published validation studies with race-stratified, ethnicity-stratified, and socioeconomic status–stratified outcomes,^68^ representing a critical evidence gap.

### Structural barriers to equitable benefit

Even when devices are accessible and algorithms are accurate, the ultimate health benefit depends on integration with responsive health care systems. Early detection of acute ischemic events through wearable monitoring is only as valuable as the clinical follow-up it enables. Populations with limited access to primary care, those without a usual source of health care, or individuals facing language or trust barriers may not experience the same benefits from early warnings.^69^ A wearable alert may generate anxiety without improving outcomes if it cannot be acted upon because of lack of insurance, inability to access emergency care, or mistrust of the health care system. Thus, wearable technologies must be implemented alongside efforts to strengthen health care access for underserved communities.

### Call for equity-focused research and development

Addressing these equity considerations requires intentional action across the technology development and validation pipeline. First, device design should prioritize affordability and inclusivity, including features that accommodate lower digital literacy or language diversity. Second, regulatory approval and clinical adoption should require evidence that algorithms perform consistently across race, ethnicity, and socioeconomic strata. Prospective validation studies must stratify results by these demographic variables to identify differential performance.^70^ Third, public health initiatives and health care systems should develop implementation strategies specifically targeting underserved patient groups, ensuring that the benefits of wearable monitoring do not accrue solely to those already privileged. Finally, researchers and manufacturers should engage with community stakeholders to understand barriers and codesign solutions that promote equitable adoption.

In summary, the promise of wearable ECG technology for ACS detection will only be fully realized if equity is embedded as a core design principle, not an afterthought. Without deliberate attention to the digital divide, algorithmic fairness, and structural barriers, these innovations risk creating a 2-tiered system where those at highest risk are left behind.

## Conclusions

The emergence of wearable ECG technology represents a pivotal shift in the management of ACSs, moving the diagnostic paradigm from intermittent, hospital-based snapshots to continuous, longitudinal surveillance. As this review has highlighted, the standard 12-lead ECG—although remaining the diagnostic gold standard—is constrained by its inability to capture transient or atypical acute ischemic events that occur during daily life before hospital arrival. Wearable devices, ranging from ubiquitous smartwatches and handheld monitors to advanced adhesive patches and smart textiles, offer a promising solution to this “diagnostic gap” by enabling real-time detection of repolarization abnormalities outside the clinical environment.

As summarized in the Central Illustration, a critical evaluation of current technologies reveals a distinct trade-off between wearability and diagnostic fidelity. Although single-lead devices, particularly smartwatches, have achieved widespread adoption and high specificity, their sensitivity for detecting ACS is inherently limited by their restricted spatial view of the myocardium. The evidence suggests that for wearable technology to reliably detect acute ischemic changes, the evolution toward multilead configurations—whether through synthesized lead reconstruction, user maneuvers, or multielectrode patch designs—is essential. Without adequate lead coverage, significant regions of the heart, particularly the inferior and posterior walls, remain electrically silent to current wearable sensors.

**Central Illustration:**
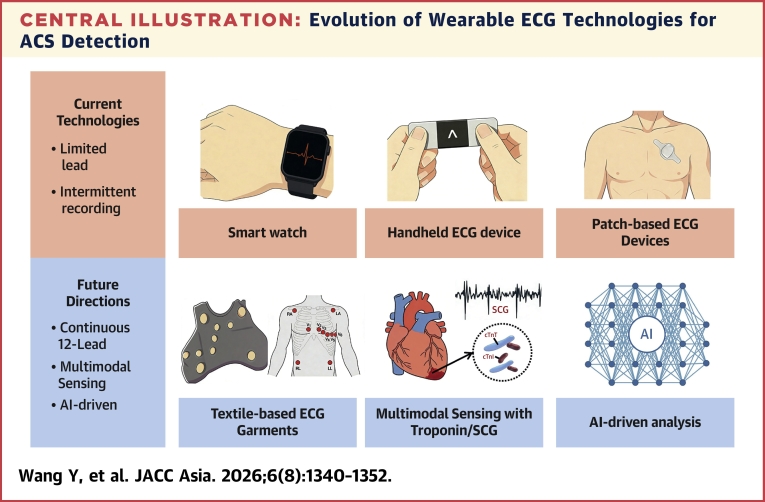
Evolution of Wearable ECG Technologies for ACS Detection This schematic contrasts current limitations of wearable ECG monitoring (limited-lead, intermittent recording) with future technological directions. Key advancements include synthesized multilead systems for enhanced spatial resolution, multimodal sensing combining ECG with troponin or seismocardiography parameters, and AI-driven personalized analysis. ACS = acute coronary syndrome; AI = artificial intelligence; ECG = electrocardiogram; SCG = seismocardiography.

Looking forward, the transformation of these devices from wellness trackers to medical-grade instruments hinges on overcoming substantial technical and clinical hurdles. The reliability of ambulatory data remains vulnerable to motion artifacts and signal degradation, necessitating the development of robust dry-electrode materials and adaptive signal processing algorithms. Furthermore, to transcend the limitations of electrophysiology alone, the future of monitoring for acute ischemic events lies in multimodal sensing—integrating ECG data with mechanical wall-motion sensors or real-time biochemical assays—and the deployment of AI to interpret the resulting complex data sets.

However, technological capability alone is insufficient to ensure clinical impact. Realizing the full potential of wearable monitoring also demands overcoming parallel challenges in implementation and health equity—including bridging the digital divide, ensuring algorithmic fairness across diverse patient groups, and seamlessly integrating alerts into accessible health care systems. Without deliberate attention to these factors, the benefits of these innovations risk exacerbating existing disparities in cardiovascular outcomes. Ultimately, the successful integration of these technologies into clinical practice requires rigorous, large-scale validation trials to establish ambulatory thresholds for acute ischemia. If these challenges can be met, wearable ECG technology stands to revolutionize cardiovascular care, enabling a proactive, personalized approach to the early detection and management of ACS.

## Funding Support and Author Disclosures

The authors have reported that they have no relationships relevant to the contents of this paper to disclose.
